# Automated processing pipeline for neonatal diffusion MRI in the developing Human Connectome Project

**DOI:** 10.1016/j.neuroimage.2018.05.064

**Published:** 2019-01-15

**Authors:** Matteo Bastiani, Jesper L.R. Andersson, Lucilio Cordero-Grande, Maria Murgasova, Jana Hutter, Anthony N. Price, Antonios Makropoulos, Sean P. Fitzgibbon, Emer Hughes, Daniel Rueckert, Suresh Victor, Mary Rutherford, A. David Edwards, Stephen M. Smith, Jacques-Donald Tournier, Joseph V. Hajnal, Saad Jbabdi, Stamatios N. Sotiropoulos

**Affiliations:** a Wellcome Centre for Integrative Neuroimaging, Oxford Centre for Functional Magnetic Resonance Imaging of the Brain (FMRIB), University of Oxford, UK; b Centre for the Developing Brain, King's College London, UK; c Department of Computing, Imperial College London, UK; d Sir Peter Mansfield Imaging Centre, School of Medicine, University of Nottingham, UK

**Keywords:** Diffusion MRI, Tractography, Quality control, Brain, Connectome, Newborn

## Abstract

The developing Human Connectome Project is set to create and make available to the scientific community a 4-dimensional map of functional and structural cerebral connectivity from 20 to 44 weeks post-menstrual age, to allow exploration of the genetic and environmental influences on brain development, and the relation between connectivity and neurocognitive function. A large set of multi-modal MRI data from fetuses and newborn infants is currently being acquired, along with genetic, clinical and developmental information. In this overview, we describe the neonatal diffusion MRI (dMRI) image processing pipeline and the structural connectivity aspect of the project. Neonatal dMRI data poses specific challenges, and standard analysis techniques used for adult data are not directly applicable. We have developed a processing pipeline that deals directly with neonatal-specific issues, such as severe motion and motion-related artefacts, small brain sizes, high brain water content and reduced anisotropy. This pipeline allows automated analysis of in-vivo dMRI data, probes tissue microstructure, reconstructs a number of major white matter tracts, and includes an automated quality control framework that identifies processing issues or inconsistencies. We here describe the pipeline and present an exemplar analysis of data from 140 infants imaged at 38–44 weeks post-menstrual age.

## Highlights

• A comprehensive and automated pipeline to consistently analyse neonatal dMRI data.
• Optimised motion and distortions correction to address newborn specific challenges.
• The automated QC framework allows to detect issues and to quantify data quality.
• Automated white matter segmentation allows to extract tract-specific masks.
• Preliminary data analysis of 140 infants imaged at 38–44 weeks post-menstrual age.

## Introduction

A key challenge in neuroscience is to understand how the human brain grows and adapts during development (Kaiser, 2017). Magnetic Resonance Imaging (MRI) and the advent of connectome mapping methods have revolutionized the way we can approach this challenge by allowing non-invasive multi-modal explorations in living subjects (Miller et al., 2016; Van Essen and Ugurbil, 2012). Connectome studies require large datasets, and to date have been most successful in defining cerebral topography (Glasser et al., 2016) or linking connectivity to behaviour in adults (Smith et al., 2015). The developing Human Connectome Project (dHCP, http://www.developingconnectome.org) is set to create and make openly available a 4-dimensional map of early structural and functional developmental changes. A large dataset of multi-modal MRI scans from fetuses and newborn infants aged 20–44 weeks post-menstrual age is currently being acquired, alongside genetic, clinical and developmental information, with the aim of allowing researchers to explore genetic and environmental influences on brain development, and to further our understanding of the relation between cerebral connectivity and neurocognitive function.

**Fig. 1:**
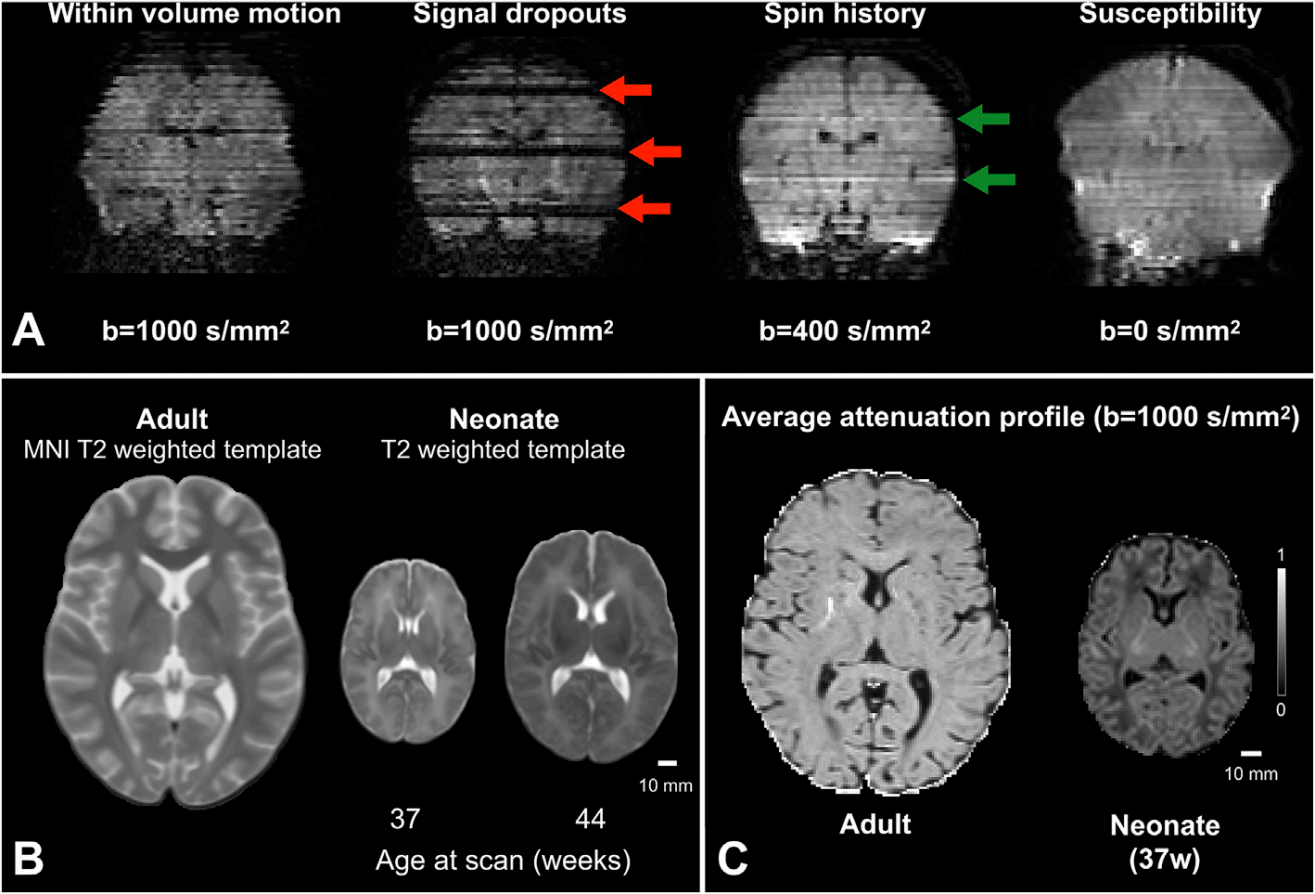
Overview of some challenges posed by neonatal dMRI data processing. A) An image damaged by motion causes slice misalignment due to within-volume motion, severe signal dropouts across multiple slices (red arrows) and signal hyper-intensities due to spin-history effects (green arrows). Moreover, when using fast EPI sequences, susceptibility induced distortions are present along the phase encoding direction. B) Changes in gyrification profiles and tissue contrast throughout development need to be accounted for when analysing neonatal data; moreover, small age differences typically correspond to significant brain volume changes, which can modulate the data SNR. C) The white matter signal attenuation (i.e., the ratio between averaged diffusion weighted and diffusion un-weighted signal) of neonates is higher and shows more contrast than in the adult brain.

In this work, we focus on newborn infants and on structural connectivity assessed using diffusion MRI (dMRI). This is a powerful technique to probe brain connections and microstructure during early development (Ball et al., 2013a, 2013b, 2015; Batalle et al., 2017b; Chung et al., 2016; Dubois et al., 2014a; Huppi and Dubois, 2006; Mishra et al., 2013; Pandit et al., 2013, 2014; Smyser et al., 2016; Yu et al., 2016). However, dMRI in newborn infants poses several challenges (Heemskerk et al., 2013; Pannek et al., 2012). Imaging newborn infants can be difficult and requires specialized medical facilities, operators, equipment and acquisition protocols (Hughes et al., 2017). Image analysis is challenging, and tools developed for adult brain data have to be refined or extended. Problems that are commonly overlooked or negligible when processing adult data, for example within-volume motion, need to be considered when analysing neonatal dMRI (Fig. 1A). Excessive motion can also cause severe signal dropouts that do not reflect the anatomically related anisotropic behaviour of water molecules; moreover, in combination with short repetition times it can cause significant intensity changes due to spin-history effects. The spin-history effects can be further aggravated by thick and overlapping slices due to the resulting reduced time between consecutive excitations of any given spin. Small and variable head sizes modulate the overall signal-to-noise ratio (SNR) and have to be accounted for (Fig. 1B). Cerebral free water content is higher in the infant brain, anisotropy is lower, and inhomogeneous maturation alters the signal profile significantly within a few weeks (Fig. 1C). Bespoke solutions are thus required for the successful connectomic analysis of neonatal dMRI.

In this work, we present a fully automated processing pipeline developed and optimised for neonatal dMRI data. The pipeline takes as input raw data and information about the scanning protocol, corrects for distortions, artefacts and subject motion, and super-resolves the originally acquired thick, but overlapping slices. The pipeline then estimates fibre orientations with methods that consider the inherently low anisotropy in white matter of the neonatal brain. These results are then used by an automated tractography framework to segment 16 (13 of which are bilateral) association, projection, limbic, cerebellar and commissural tracts in age-matched template space (Serag et al., 2012). This allows mapping tract-specific microstructural and morphological changes at the population level. Given the large number of subjects in the dHCP, an automated quality control (QC) framework has been implemented to assess quality and allow the detection of residual issues or inconsistencies with the data. The proposed data processing is applied to an exemplar dataset of infants imaged at approximately term-equivalent age.

## Subjects and dMRI acquisition

MR images were acquired as a part of the dHCP; the study was approved by the National Research Ethics Committee and informed written consent given by the parents of all participants. Infants were fed and wrapped and encouraged to sleep naturally in the scanner; no sedation was administered. Images were acquired in the Evelina Newborn Imaging Centre, located within the Neonatal Intensive Care Unit of the Evelina London Children's Hospital. Pulse oximetry, temperature and heart rate were monitored throughout and ear protection was provided for each infant (President Putty, Coltene Whaledent, Mahwah, NJ; MiniMuffs, Natus Medical Inc., San Carlos, CA), and a paediatrician or neonatal nurse present throughout the scan. A dedicated patient handling system has been developed (Hughes et al., 2017) which encourages natural sleep and reduces the propensity of the infant to move during image acquisition.

In this exemplar study the images analysed were obtained from 140 infants (51 male, 89 female) born at median (range) 40 (36.6–42.1) and imaged at 40.9 (37.6–44.4) weeks post-menstrual age (Sup. Fig. 1). All images were reviewed by a paediatric neuroradiologist prior to inclusion in the dataset.

## Preprocessing pipeline

### Acquisition protocol summary

A highly optimised and efficient dMRI sequence was implemented on a 3T Philips Achieva scanner equipped with a dedicated 32-channel neonatal head coil and 80 mT/m gradients (Hutter et al., 2017). Briefly, data were acquired with a monopolar spin echo echo-planar imaging (SE-EPI) Stejksal-Tanner sequence (Δ  = 42.5 ms, δ = 14 ms). To keep the dMRI acquisition time shorter than 20 min, a multiband factor of 4 (TR = 3800 ms, TE = 90 ms) was used. A total of 64 interleaved (step 3) overlapping slices (in-plane resolution = 1.5 mm, thickness = 3 mm, overlap = 1.5 mm) were acquired for each single volume to obtain an isotropic voxel resolution of 1.5 mm after super-resolution. The total imaged volume size was set to 150 × 150 × 96 mm^3^ to include up to the 95^th^ percentile of newborns head size distribution sampled at the 44^th^ week post-menstrual age. A total of 300 volumes (20 b0s) was collected for each subject, sampled using 4 different phase encoding (PE) directions (Posterior- > Anterior, Anterior- > Posterior, Left- > Right, Right- > Left) and 3 different b-value shells (400, 1000 and 2600 s/mm^2^). Unique diffusion encoding orientations (64, 88 and 128 per b-value shell) and magnitudes were chosen to maximise the dMRI tissue contrast between white and grey matter in neonates (Tournier et al., 2015; Tournier et al., 2018, under revision). The multi-shell HARDI protocol optimisation procedure was based on the information content of the dMRI signal, with no reliance on any specific reconstruction algorithm. Slices, diffusion encoding orientations and PE directions were optimally interleaved to limit motion artefacts and gradient duty cycle. More details on the optimisation strategy are given in (Hutter et al., 2017).

**Fig. 2:**
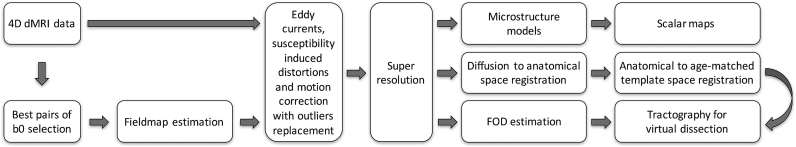
Summary overview of the dHCP neonatal processing pipeline. The “best” (i.e., the least affected by within-volume motion) pairs of b0 volumes for each phase encoding direction are extracted from the raw dMRI data. These are used to estimate the off-resonance field that is then used in the simultaneous correction of motion artefacts, susceptibility-induced and eddy current distortions. Data are super-resolved and, after pre-processing is complete, local diffusion and microstructural models are fitted in every voxel. After registration of dMRI data to the age-matched template automated tractography extracts a number of white matter tracts.

Fig. 2 shows a summary of the automated dHCP neonatal diffusion pipeline. In the following sections, we detail each processing stage and discuss the approaches used to deal with specific challenges.

### Off-resonance field estimation

A fast SE-EPI sequence is used to minimise the effect of motion. However, EPI is very sensitive to susceptibility differences at tissue interfaces which create field inhomogeneities that lead to image distortions along the PE direction.

To correct for such susceptibility-induced distortions, one could measure the off-resonance field directly (Jezzard and Balaban, 1995) or, as in the dHCP neonatal acquisition protocol (Hutter et al., 2017), acquire the dMRI data using different PE directions (Andersson et al., 2003). More specifically, combining EPI images acquired using reverse PE directions provides information about susceptibility-induced distortions. This information can then be used to estimate the susceptibility-induced off-resonance field that, when converted to a displacement field and applied to the raw data, can recover the correct location of each distorted voxel and restore the correct brain geometry. This is of primary importance when trying to register dMRI data to a higher resolution anatomical volume (e.g. T1 or T2-weighted).

In the dHCP, 4 PE directions were acquired. When estimating the susceptibility field from multiple volumes with different PE-encoding it is important that those volumes are unaffected by intra-volume movement. Therefore, to avoid motion-induced signal dropouts the pipeline selects the “best” pair of b0 volumes (i.e., the least affected by apparent shape changes due to intra-volume subject motion) for each PE direction (Alfaro-Almagro et al., 2017). To do this, all b0 volumes for each separate PE direction are first aligned using a rigid-body registration (6 degrees of freedom) (Jenkinson et al., 2002). Then, the average correlations between each b0 volume and the other PE-matched ones are obtained. The two b0 volumes for each PE direction with the highest average correlation are selected as they are less affected by intra-volume motion. The volume with the highest mean correlation to all other b0s is selected as the “reference” volume for subsequent pre-processing (Sup. Fig. 2).

**Fig. 3:**
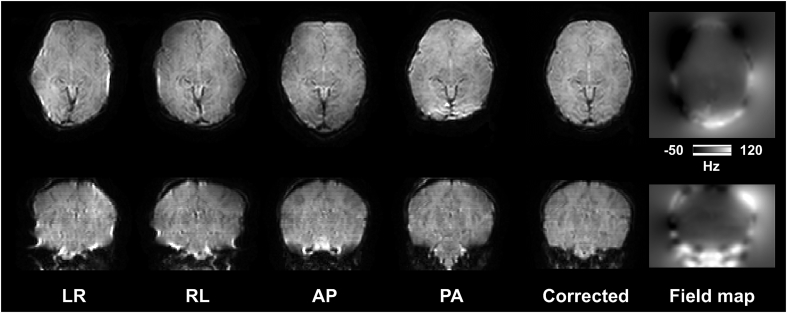
Raw b0 volumes acquired using four different PE directions (LR: Left - > Right, RL: Right - > Left, AP: Anterior - > Posterior, PA: Posterior - > Anterior). Fifth column shows an example volume after correcting for susceptibility-induced distortions. Last column shows the estimated field map in Hertz.

Fig. 3 shows typical raw and corrected b0 images acquired using four PE directions on a newborn infant imaged at 41 weeks GA. Different volumes show different amount and direction of susceptibility-distortions, depending on which PE direction was used during their acquisition. We use FSL's TOPUP (Andersson et al., 2003; Smith et al., 2004) to estimate the off-resonance field, which is then used in the next step (see below) where susceptibility- and eddy current-induced distortions are corrected along with subject movement.

### Distortion correction (susceptibility, eddy currents and motion)

To address distortions caused by motion, motion-induced signal drop-out and eddy currents, we used a non-parametric approach (FSL EDDY) which corrects for these distortions in a single resampling step (Andersson and Sotiropoulos, 2016). This framework is based on a Gaussian Process (GP) predictor of undistorted data (Andersson and Sotiropoulos, 2015), which exploits redundancies in the input data. Volumes sampled using diffusion-sensitisation directions lying within opposing cones on the sphere exhibit opposite eddy-current distortions and therefore their average is approximately distortion-free. A GP uses as an input the whole measured dataset to represent how the shape of the diffusion signal varies as a function of b-value and diffusion encoding orientation and generates predictions in a distortion-free space. GP-based measurement predictions are compared with the data and the difference between the two is used to update the current estimates of the eddy currents and motion parameters. The estimates are iteratively refined until convergence and once estimated they are used to undistort the original data. At the end of the iterative process, the motion parameters are also used to rotate the user-provided diffusion encoding orientations (Leemans and Jones, 2009).

**Fig. 4:**
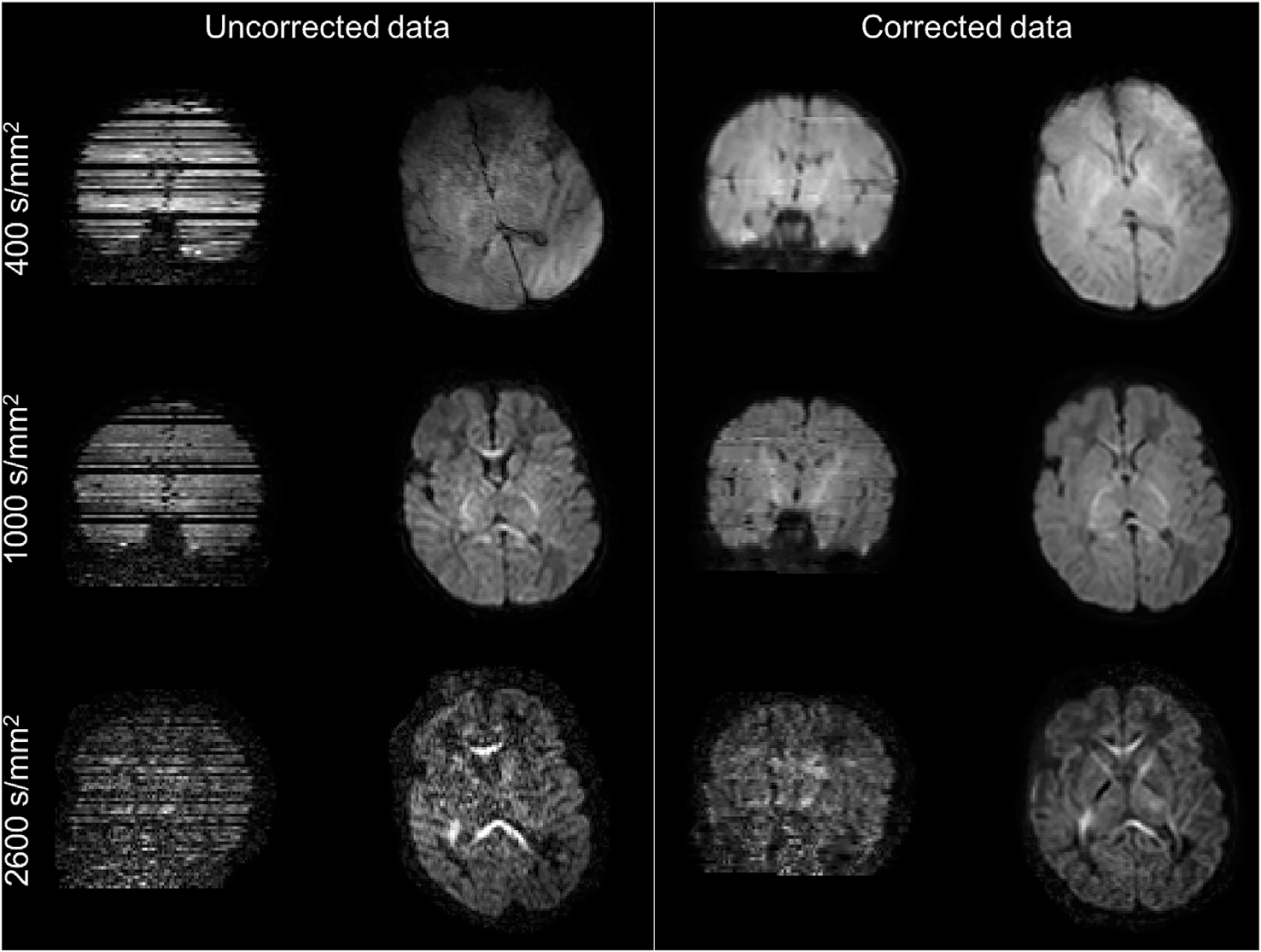
Coronal and axial views of raw and pre-processed dMRI images using the outlier identification and replacement extension of EDDY. The data has been corrected for motion, susceptibility and eddy-current-induced distortions, and slices affected by severe signal dropouts have been correctly identified as outliers and replaced with the predicted signal in undistorted space.

Recent extensions to this framework allow outlier slice detection and replacement (Andersson et al., 2016). It detects signal dropouts caused by excessive motion present in one or more slices (Fig. 4), by comparing the observed signal to the predictions from the GP. When an outlier has been detected it is discarded, a new prediction is made (without the outlier) and used to replace the outlier slice (Andersson et al., 2016). We set a threshold of 4 standard deviations away from the predicted intensity to detect outlier slices. Fig. 4 shows the result of such outlier detection and replacement approach on a neonatal dHCP subject for all the three acquired b-shells. Susceptibility-induced and eddy-current distortions have been corrected, and the slices affected by severe dropouts correctly identified and replaced with the predictions of the distortion correction framework. This replacement allows subsequent microstructure estimates to be unbiased by the non-biologically relevant drop in the signal (Andersson et al., 2016) and also permits unbiased registration of diffusion to structural data.

**Fig. 5:**
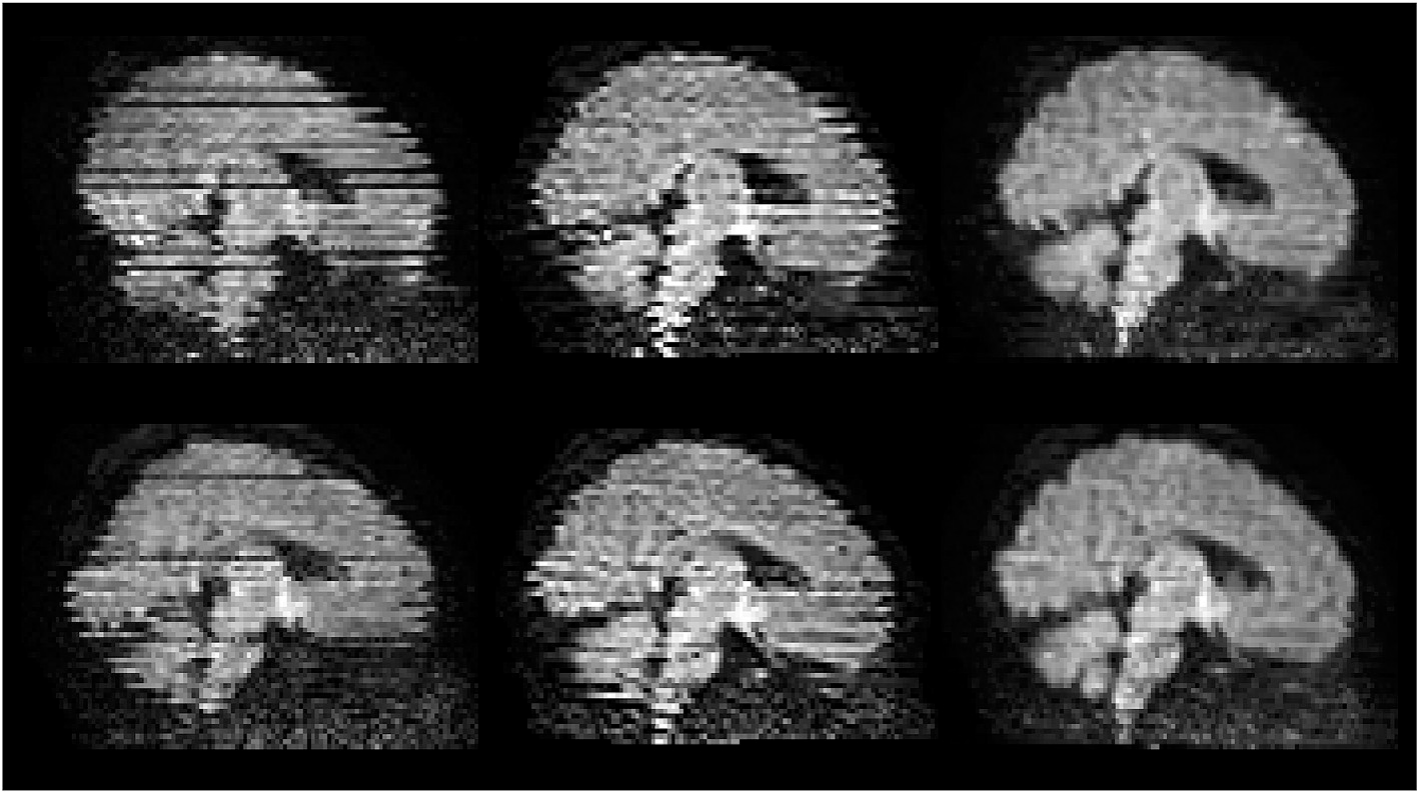
The leftmost column shows sagittal views from two volumes (b = 1000 s/mm^2^) of one subject. The middle and rightmost columns show pre-processed sagittal views of the same two volumes using a volume-based and a slice-to-volume movement model respectively.

A second extension deals with intra-volume motion, by realigning slices within the same volume. The slice-to-volume movement model (Andersson et al., 2017; Kainz et al., 2015) augments the temporal resolution at which movement is estimated, which is originally constrained by the repetition time (TR) by fitting a continuous movement model. Fig. 5 shows the results when correcting for intra-volume motion on an example dHCP subject who was moving significantly during the acquisition of individual dMRI volumes. The figure shows that, when only a volume-based approach is used, a tell-tale zig-zag outline is still present and that this is corrected when using the proposed approach.

### Super resolution

The optimised dHCP imaging protocol (Hutter et al., 2017) aims at acquiring a high number of volumes (i.e., 300 in total) in the shortest possible time. To achieve maximum brain coverage in the presence of motion while retaining a high SNR, the slice thickness has been set to double the in-plane resolution, i.e., 3 mm, while keeping the slice spacing at 1.5 mm. As the number of samples is the same as for the isotropic resolution 1.5 mm, a super-resolution reconstruction algorithm (Greenspan, 2009; Kuklisova-Murgasova et al., 2012) can be applied to recover isotropic resolution on a regular grid. The use of thicker overlapping slices is beneficial in avoiding data gaps when there is subject motion, but it can also aggravate spin-history effects and result in blurring in the through-plane direction. Super-resolution algorithms remove this blurring by modelling the acquisition of thick slices from a high-resolution isotropic volume through a known point spread function (PSF) and solving the inverse problem to recover the isotropic volume from the acquired thick-slice data. In the current pipeline the super-resolution algorithm is applied to the distortion-corrected data and separately to each diffusion weighted volume along the slice-selection direction to prevent the computational burden of using the whole dataset simultaneously.

### Registration to anatomical and standard space

**Fig. 6:**
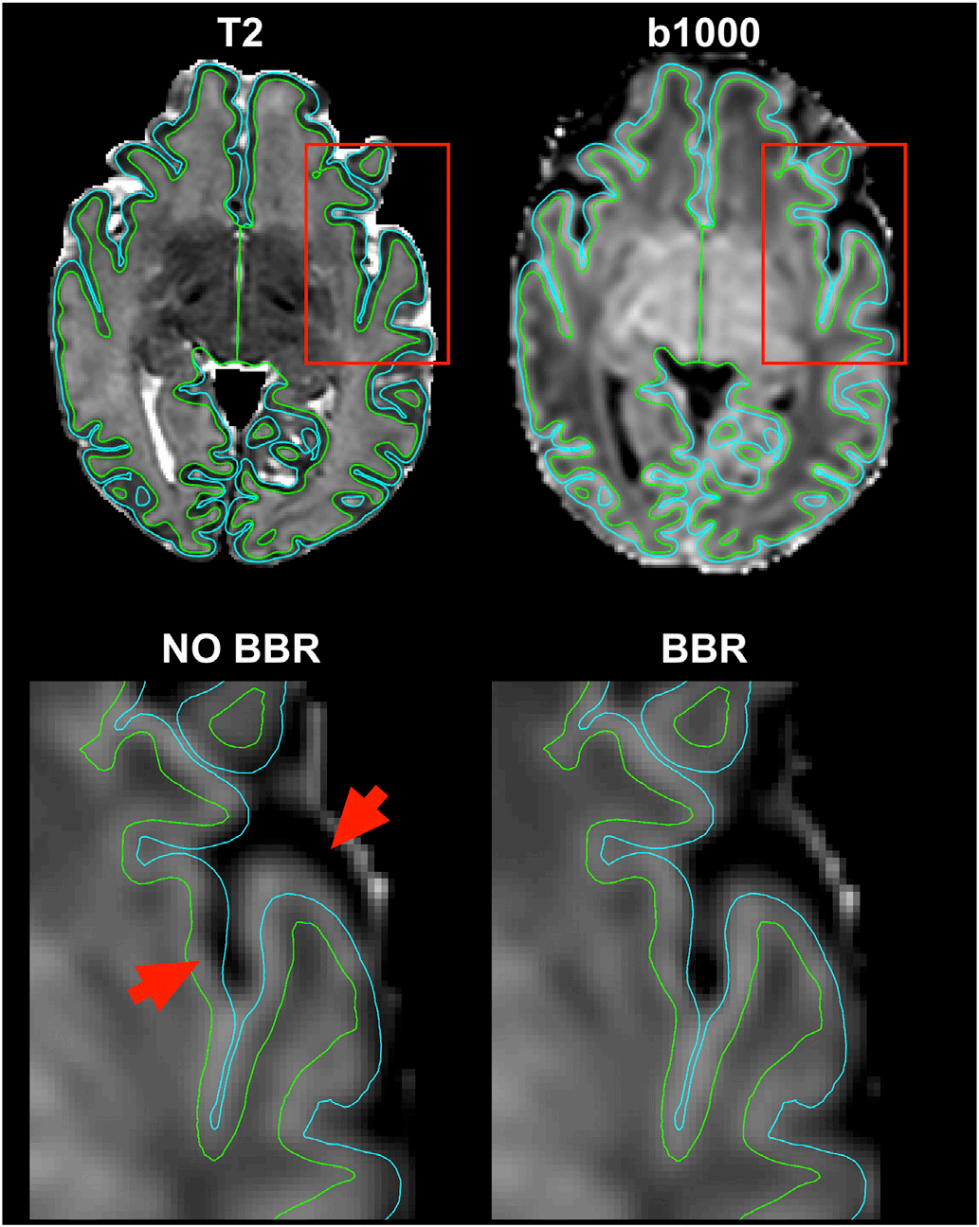
Registration accuracy using BBR. The fully pre-processed average attenuation profile volume for the b = 1000 s/mm^2^ shell is registered to the high resolution T2-weighted volume in subject's native space (top row). When using BBR the tissue boundaries are better aligned to those extracted from the high-resolution volume, as shown by the two insets (red box). Red arrows point to areas where standard volumetric registration resulted in residual misalignments in the left hemisphere (bottom row). White/grey matter (green) and grey/csf boundaries (light blue) are overlaid on top of the attenuation profile transformed to the T2 anatomical space.

Transformations that align diffusion data to structural images are obtained to allow further registration between subjects and to templates. Because of its high SNR and contrast between white and cortical grey matter, the average attenuation profile of the b = 1000 s/mm^2^ shell is used for registration of diffusion data to the high-resolution T2-weighted volume. We use FLIRT (Jenkinson et al., 2002) to perform a rigid body transformation with 6 degrees of freedom (DOF) with a boundary-based-registration (BBR) cost function as described in (Greve and Fischl, 2009). BBR offers the advantage of being less sensitive to post-processing residual distortions, compared to volumetric registration approaches (Fig. 6). When compared to the results obtained using raw unprocessed data, we observed very low correlation values for some subjects. These were caused by misregistrations of the raw data to anatomical space due to very low tissue contrast and high distortions (Sup. Fig. 3). We then combine the linear transformation matrix (diffusion to T2) with a non-linear warp registration (T2 to age-matched template) field using FSL FNIRT (Andersson et al., 2010) to obtain transformations that map from diffusion space to an age-matched template (Serag et al., 2012). We use these transformations to average scalar maps and fibre orientations (after applying the appropriate rotations) across subjects in age-matched template space. We obtain the white matter volume segmentation necessary for BBR and the non-linear warp fields from the dHCP structural pipeline results (Makropoulos et al., 2017). Lastly, the resulting transformation is then also inverted to move from age-matched template space to the subject's diffusion space.

## Quality control

When dealing with large datasets such as the dHCP, automated quality control (QC) is of great importance for detecting data processing issues and inconsistencies. We have developed a dMRI QC framework that reads the outputs at different stages of the processing pipeline and generates summary data quality statistics (Bastiani et al., submitted). QC is performed at both the subject and the group level. Table 1 gives an overview of all the estimated QC indices.

**Table 1:** Overview of QC indices.

Processing stage		
Data import	Distortions and motion correction	Registration to anatomical space
Acquired volumes (%)	Average absolute motion (mm)	T2w-b0 correlation
	Average relative motion (mm)	Average white matter CNR
	Total outliers (%)	Average white matter SNR
	Outliers per b-shell (%)	Average grey matter SNR
	Outliers per PE direction (%)	Average white matter squared residuals
	Outliers per volume (%)	White/grey matter boundary (image)
	Outliers per slice (%)	
	Average signal profile per b-shell (image)	

At the data import stage the automated QC framework checks whether all 300 expected volumes have been acquired. The data are considered incomplete and not processed further when less than 11.3% (i.e., 34 volumes) of the total expected number of volumes have been acquired. This is the minimum number of volumes we need to be able to estimate an off-resonance field as two b0 volumes with opposite PE directions are acquired when reaching 34 volumes. If this threshold is reached, we are able to run through the distortion correction step and generate QC metrics. It does not imply optimality in any sense and the reduced number of volumes will be reflected in reduced values of all CNR metrics.

Absolute and relative motion are computed and reported. The former quantifies the displacement between the reference volume and each of the other volumes, the latter the displacement between two consecutive dMRI volumes. We found that for dHCP subjects born at term, the median across subjects of average absolute motion was 1.52 mm (std: 0.62, min: 0.56, max: 6.31 mm), i.e., slightly more than a voxel. As a comparison, we computed the average absolute within-session motion for a sub-set (n = 50) of subjects from the adult Human Connectome Project (HCP) and found a median of 0.83 mm (std: 0.29, min: 0.11, max: 3.21 mm).

Several outlier-related QC metrics are computed. Total number of outliers are split according to b-value shell, PE direction and single volume. The number of times a single slice has been marked as outlier is also computed to check for stationary spatial inconsistencies in the acquisition due to image reconstruction issues or hardware faults. When compared to HCP data, the median percentage of total outlier slices is almost 5 times higher (1.89% for dHCP, 0.39% for HCP).

To check the accuracy of susceptibility-induced distortion corrections and of the registration from dMRI space to subject native anatomical space, we also compute the correlation across brain masked voxels between the registered average b0 volume and the high-resolution T2-weigthed volume. The distribution of these correlation values helps in identifying problematic subjects.

**Fig. 7:**
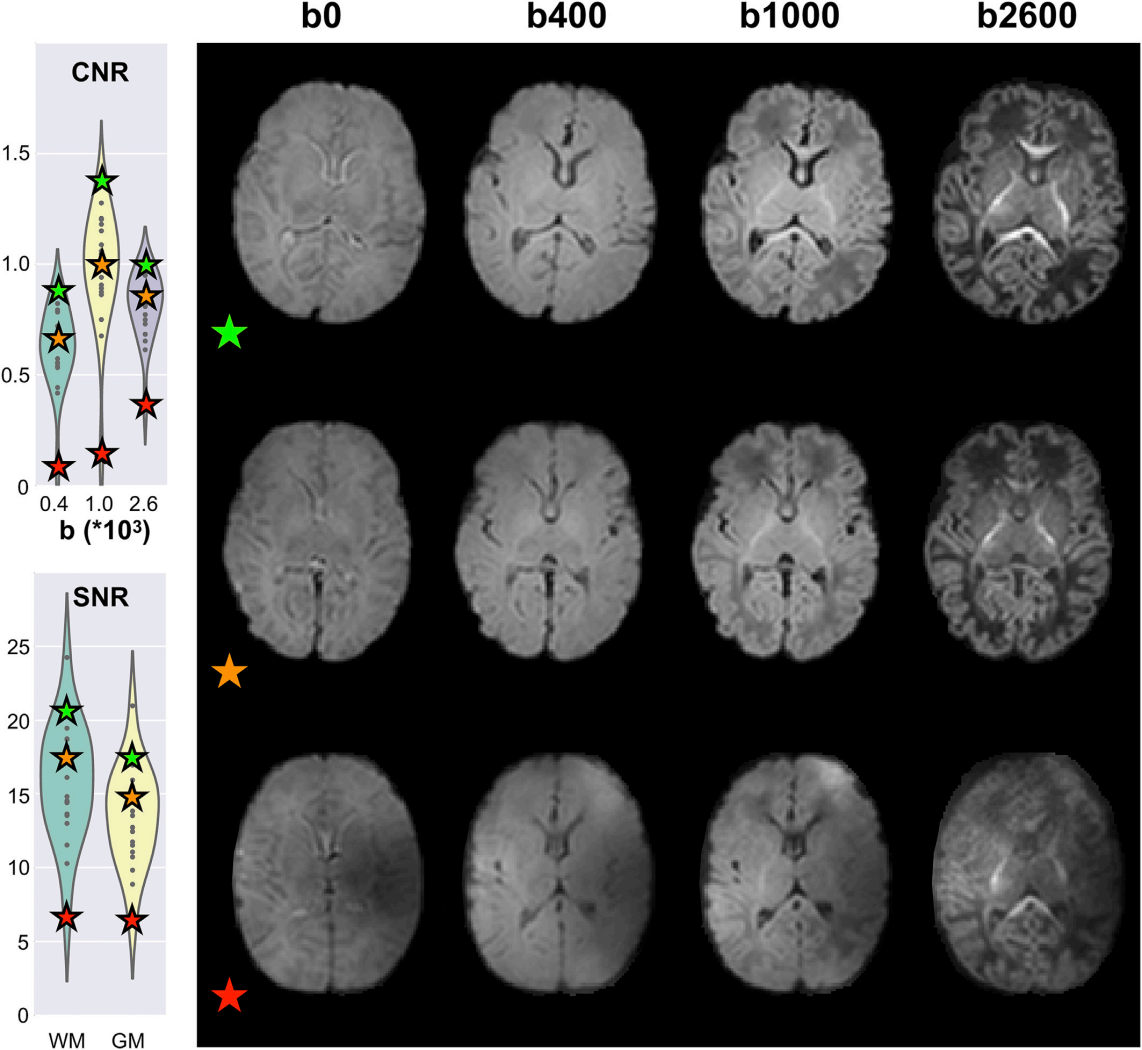
Comparison between three subjects (age-matched: 41 weeks post-menstrual age) based on average tissue-specific CNR and SNR. SNR was sampled separately from the whole white and grey matter mask, CNR was sampled only from the whole white matter mask. Violin plots show CNR and SNR group distributions for 20 age-matched subjects. The colour-coded stars represent the three example subjects. Axial slices are obtained from the respective averaged b-shell volumes. Contrast between the three main tissue types (white, grey matter and CSF) increases from the bottom to the top together with CNR and SNR estimated indices. The QC framework identifies cases where the pre-processing pipeline or data acquisition were not fully successful (bottom row). The best subject shows clear delineation between white and grey matter over the whole brain volume.

Once registration to the subject's native anatomical space is complete, white and grey matter segmented binary masks are brought to dMRI data space. These are used to compute tissue-specific average contrast-to-noise ratios (CNRs) and SNRs. We use the data predictions obtained from the non-parametric Gaussian process embedded in the EDDY tool, evaluated using the final estimates of distortion fields, to compute CNR and SNR. Specifically, we obtain the voxel-wise CNR by dividing the standard deviation of the predicted signals by the standard deviation of the residuals (i.e., the difference between raw and predicted data) in undistorted space. We obtain the CNR separately for each acquired shell. Voxel-wise SNR is computed using only the b0 volumes as the ratio between the average predicted signal and the standard deviation of the residuals in undistorted space. The voxel-wise values are then averaged within WM/GM. Both CNR and SNR are useful to quantify the quality of a dataset (Fig. 7). For 140 dHCP subjects, the median CNR for the b = 1000 s/mm^2^ shell in white matter is approximately 1.0 (std: 0.19, min: 0.12, max: 1.36), which compares to 0.45 (std: 0.2, min: 0.11, max: 0.73) for 50 HCP subjects. For 140 dHCP subjects, the median SNR in white matter is approximately 16 (std: 3.36, min: 6.65, max: 26.87), which compares to 10 (std: 1.2, min: 7.2, max: 11.3) for 50 HCP subjects. Differences in voxel size, though, play a role here as the acquired voxel volume for the dHCP is ∼3.5 times higher than for the HCP.

To investigate any potential differences or issues, we have also processed 25 extra preterm brains (age at scan range: 29–35 weeks). After running the full pipeline, we used the automated QC framework to assess differences in the distributions of quality metrics. No significant differences that would point to potential issues when processing such data were found (Sup. Fig. 4).

## Microstructure modelling

**Fig. 8:**
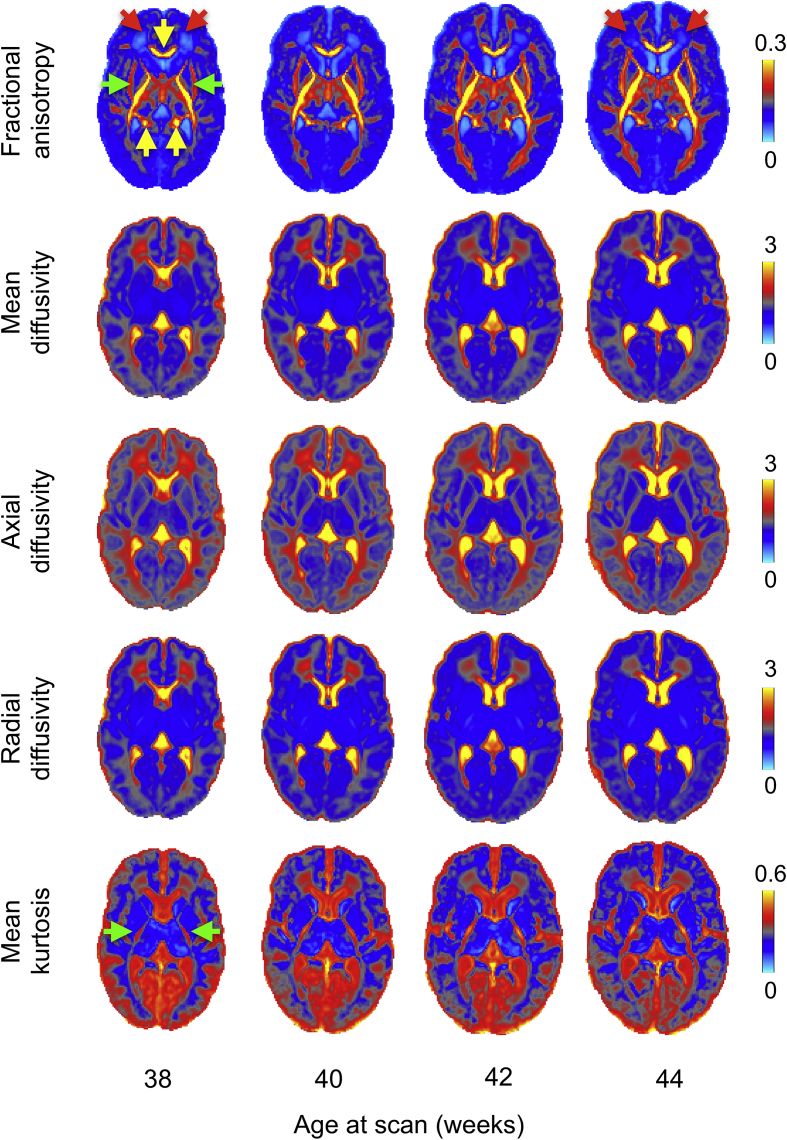
Output of the dMRI processing pipeline showing average DT-derived microstructural measures from 38 to 44 weeks (20 subjects per-age group). Expected changes with increasing age include: FA increases and MD decreases in the frontal white matter (red arrows) and internal capsules (green arrows); the corpus callosum (yellow arrows) and internal capsule both show high FA values; RD decreases; and MK declines in grey matter and increases in primary projections (green arrows). Diffusivities are expressed in μm^2^/ms.

The diffusion tensor (DT) model (Basser and Jones, 2002; Basser and Pierpaoli, 1996) is still the most widely used method to probe the brain's microstructure. However, the Gaussian displacement assumption underlying DTI breaks at high b-values. To overcome this limitation and account for deviations from Gaussian displacement, we use a diffusion kurtosis-augmented (DKI) tensor model (Jensen et al., 2005; Paydar et al., 2014). A single parameter is added to model mean kurtosis using a 2^nd^ order Taylor expansion of the log-attenuation in powers of b-values. This approach improves the overall fit to the multi-shell data and estimates a mean Kurtosis map (Fig. 8) that quantifies the deviation from Gaussianity of water molecules' displacement and can reflect different degrees of tissue heterogeneity (Steven et al., 2014). From the 6 independent components of the DT we derive the eigenvalues and eigenvectors and generate fractional anisotropy (FA), axial (AD), radial (RD) and mean diffusivity (MD) maps. Fig. 8 shows a longitudinal atlas of the estimated DT-derived microstructure parameters at 38–44 weeks post-menstrual age, with 20 subjects per age-bin (see Sup. Fig. 5 for single subjects' results).

**Fig. 9:**
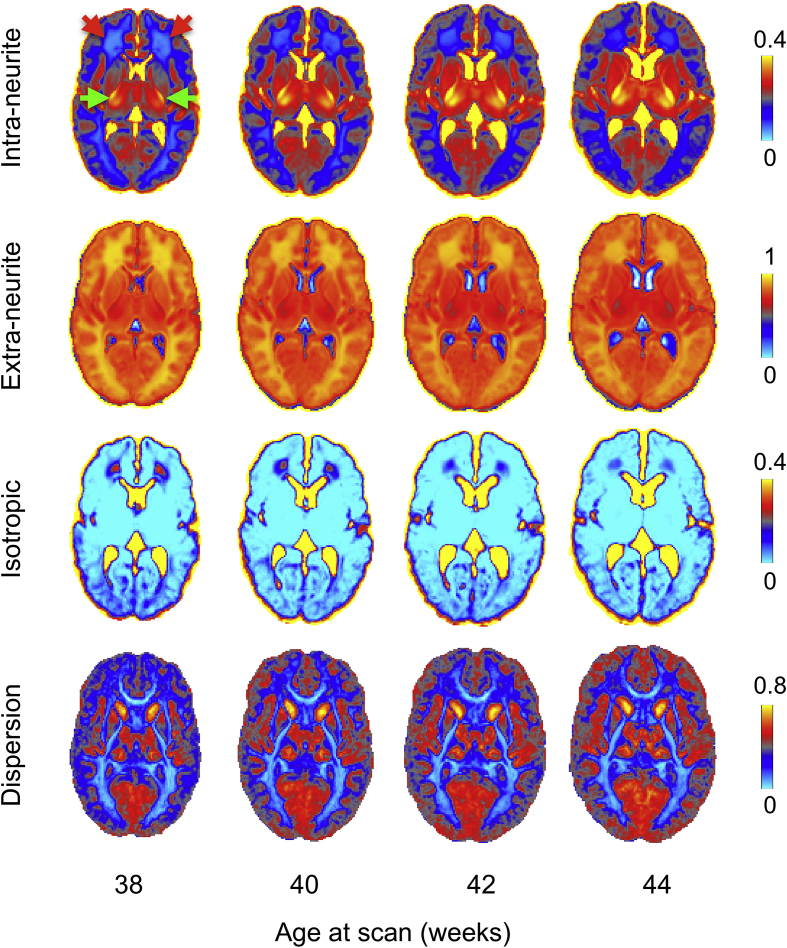
Output of the dMRI processing pipeline showing NODDI metrics from 38 to 44 weeks post-menstrual age (20 subjects per-age group). Extra-neurite volume fraction is defined as 1-(intra-neurite). Periventricular deep white matter regions (red arrows) show a high isotropic compartment volume fraction that decreases with age. Fibre dispersion also decreases in those regions. The posterior limb of the internal capsule (green arrows) shows very high intra-neurite volume fractions and low dispersion values.

We also apply the neurite orientation dispersion and density imaging (NODDI) model (Tariq et al., 2016; Zhang et al., 2012). NODDI describes the dMRI signal as a summation of three different contributing pools of water, i.e., restricted (intra-neurite), hindered (extra-neurite) and isotropic and estimates an orientation dispersion index to account for fibre fanning (Fig. 9 and Sup. Fig. 6 for single subjects' results). To model the distribution of fibre orientations it uses either Watson or Bingham distributions. We use the Bingham distribution as it allows extra flexibility by describing fibre dispersion along two orthogonal axes (Tariq et al., 2016). By introducing the orientation dispersion index and estimating the volume fractions of the intra- and extra-neurite compartments, NODDI can model tissue microstructure and geometry that may alter during development. Due to the very long computation times required to fit such a model, we use a GPU-based library for accelerated nonlinear optimisation (Hernandez Fernandez et al., 2017, www.fmrib.ox.ac.uk/∼moisesf/cudimot).

## Fibre orientation modelling

In addition to microstructure modelling, we also estimate the fibre orientation densities (FODs) that will be used for tractography. We perform the estimation using a parametric framework, based on the assumption that the voxel-wise dMRI signal is the convolution of the FOD and a single-fibre response function (Behrens et al., 2003; Tournier et al., 2004).

Differences in microstructural properties across tracts, for instance between projection and association white matter tracts, together with axonal pruning and glia development (Dehaene-Lambertz and Spelke, 2015; Dubois et al., 2008, 2014a; Huang et al., 2006; Lebel and Deoni, 2018; Mukherjee et al., 2002; Oishi et al., 2013; Wozniak and Lim, 2006), make the use of a single common single fibre response function sub-optimal. We perform parametric deconvolution using a multi-compartment model that treats the FOD as a sum of delta functions (Behrens et al., 2007; Jbabdi et al., 2012; Sotiropoulos et al., 2016). Compared to previous parametric models that use an infinitely anisotropic (stick) single-fibre response model, we use a wider profile using the response from an axially symmetric tensor (i.e. with equal second and third eigenvalues). This extension captures better the inherently low anisotropy of neonatal dMRI data. We allow changes in the anisotropy of this single-fibre response kernel (Sotiropoulos et al., 2016), as its width (λ_R_, the ratio of the minor and major axis of the tensor) is a model parameter which is estimated. We provide prior knowledge for this parameter, using a Gaussian prior for λ_R_, with the prior mean and standard deviation being estimated from the data. To do that, we sample from a white matter region where one coherent fibre population is expected. Specifically, we used a standard mask of the corpus callosum and retained, for each subject, the 50% voxels with highest FA values. We then compute the mean and standard deviation of the voxel-wise ratio λ_R_ across subjects. In the exemplar dataset of 140 infants the average λ_R_ of 0.3 and an average standard deviation of 0.08; values respectively 2.3 and 2.7 times larger than those estimated for HCP subjects (Sotiropoulos et al., 2016), reflecting the lower anisotropy in white matter.

This parametric model allows voxel-specific single-fibre response kernels (all informed by the same prior) and can estimate fibre crossings in voxels within deep white matter regions that show low degrees of anisotropy. To estimate all the different parameters and their uncertainties, we use Bayesian inference and a Rician noise model. The estimation framework is capable of removing unnecessary fibre orientations by using automatic relevance determination (ARD) priors, as in (Behrens et al., 2007).

**Fig. 10:**
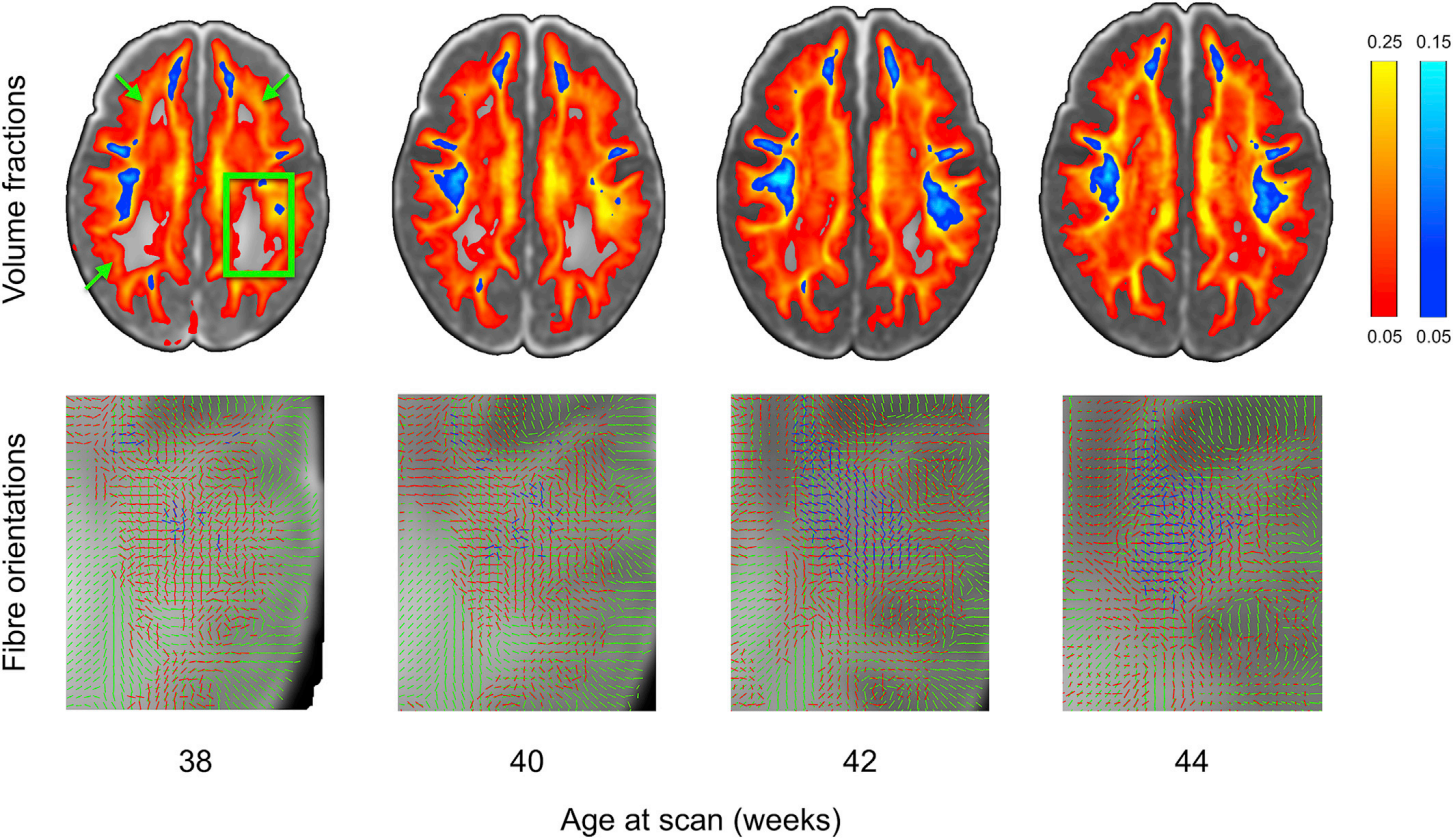
Output of the dMRI processing pipeline from 38 to 44 weeks post-menstrual age (20 subjects per bin) showing changes in fibre orientation estimations. Upper panel shows volume fractions of the 2^nd^ (red-yellow) and 3^rd^ (blue-light blue) fibres averaged across bin overlaid on top of the T2-weighted template. Second fibres get increasingly estimated across age in the periventricular deep white matter regions (green arrows). In the highlighted posterior portion of the centrum semiovale region (green box, zoomed insets in the bottom row) up to three fibres are estimated across age and survive the .05 threshold on their average volume fractions (1^st^ fibre in green, 2^nd^ in red and 3^rd^ in blue).

Fig. 10 shows averaged volume fraction maps of secondary and tertiary fibre orientations from 38 to 44 weeks post-menstrual age, where 2^nd^ and 3^rd^ fibre populations become more prominent over time, particularly in regions where adult brain shows multiple fibre populations crossing such as periventricular deep white matter regions. The figure shows the results obtained from a model selection procedure (ARD; see Behrens et al., 2007). This method automatically rejects extra fibre compartments that are not strongly supported by the data. Therefore, because those regions show a very low diffusion contrast-to-noise ratio (CNR) due to low fibre coherence and/or high water content, it is very challenging to reliably estimate more than one fibre compartment. The increased ability to detect crossings at older ages is indicative of higher diffusion contrast in the data, caused by higher structural coherence and myelination in white matter. This is probably reflecting the well-known increase of myelination that reduces the extra-axonal water fraction and the development of short cortico-cortical projections (Takahashi et al., 2012; Xu et al., 2014).

## Tractography

Given the significant structural connectivity changes that occur in the neonatal brains, it is of interest to build an atlas of white matter connections, standardized across different developmental stages. We have developed standard-space tractography protocols that allow to automatically reconstruct 16 white matter tracts (see Table 2) across subjects and across different ages.

**Table 2:** List of automatically virtually dissected fibres. Protocols comprising different ROIs are available to obtain all these tracts in age-matched space. When needed, homologues ROIs are defined in both hemispheres.

Category	Name	Bilateral
Projection fibres	Corticospinal tract (cst)	X
	Acoustic radiation (ar)	X
	Anterior thalamic radiation (atr)	X
	Superior thalamic radiation (str)	X
	Posterior thalamic radiation (ptr)	X
Association fibres	Superior longitudinal fasciculus (slf)	X
	Inferior longitudinal fasciculus (ilf)	X
	Inferior fronto-occipital fasciculus (ifo)	X
	Uncinate fasciculus (unc)	X
Callosal fibres	Forceps minor (fmi)	
	Forceps major (fma)	
Cerebellar fibres	Middle cerebellar peduncle (mcp)	
	Medial lemniscus (ml)	X
Limbic system fibres	Fornix (fx)	X
	Cingulate gyrus part of the cingulum (cgc)	X
	Parahippocampal part of the cingulum (cgh)	X

**Fig. 11:**
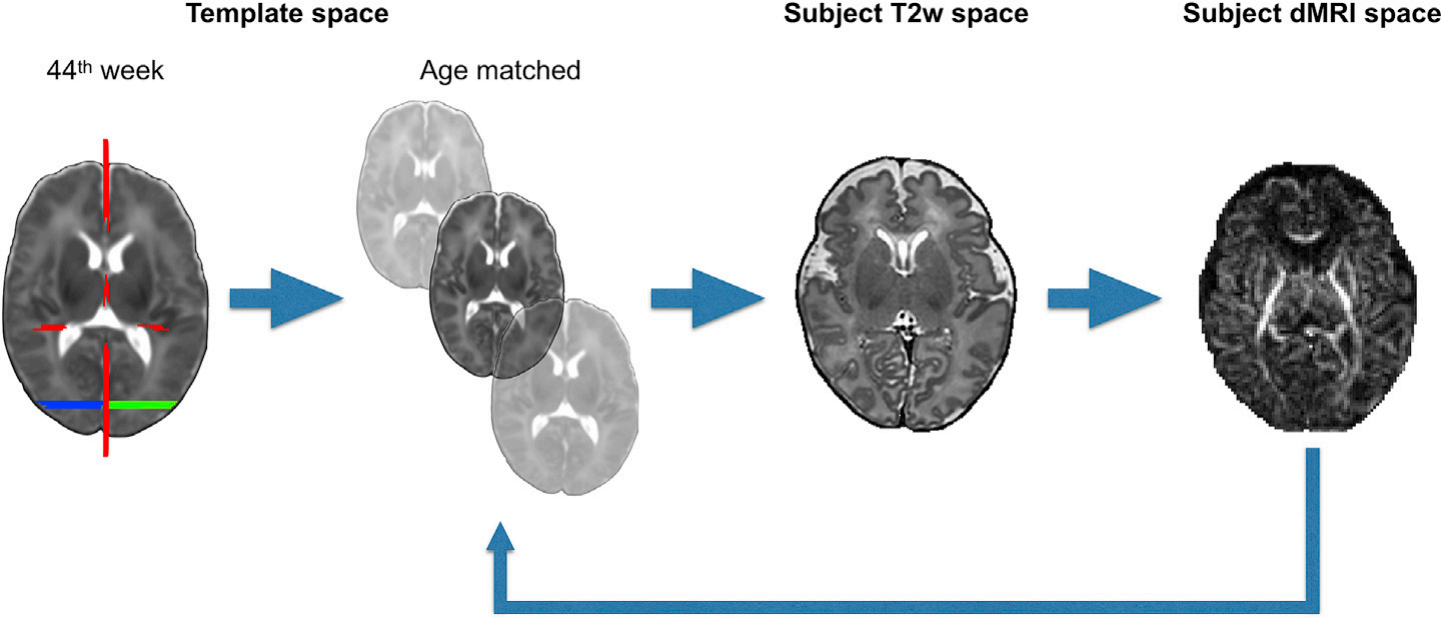
Automated probabilistic tractography framework. A set of standardized ROIs is defined for week 44. The example shows the masks for virtually dissecting the forceps major: seed mask is in green, target mask is in blue and exclusion mask is in red. The ROIs are then warped first from week 44 to the age-at-scan-matched template and then to the subject's dMRI space. Tractography is run in subject diffusion space and results are then stored in age-at-scan-matched template space.

Specifically, we defined a set of standardized ROIs in template space (Serag et al., 2012) for week 44, i.e., the available time point closest to adult age (Fig. 11). Based on white matter atlases (Catani and Thiebaut de Schotten, 2008; Wakana et al., 2004) and using the segmentation results from the dHCP structural pipeline, we defined sets of “seed” (regions where tracts start), “waypoint” (regions through which tracts should go through) and “stop” (regions where tracts terminate) masks for each tract (de Groot et al., 2013). “Exclusion” (regions that tracts should avoid) masks were also used for all tracts, comprising at least the whole mid-sagittal plane, except for the commissural projections.

**Fig. 12:**
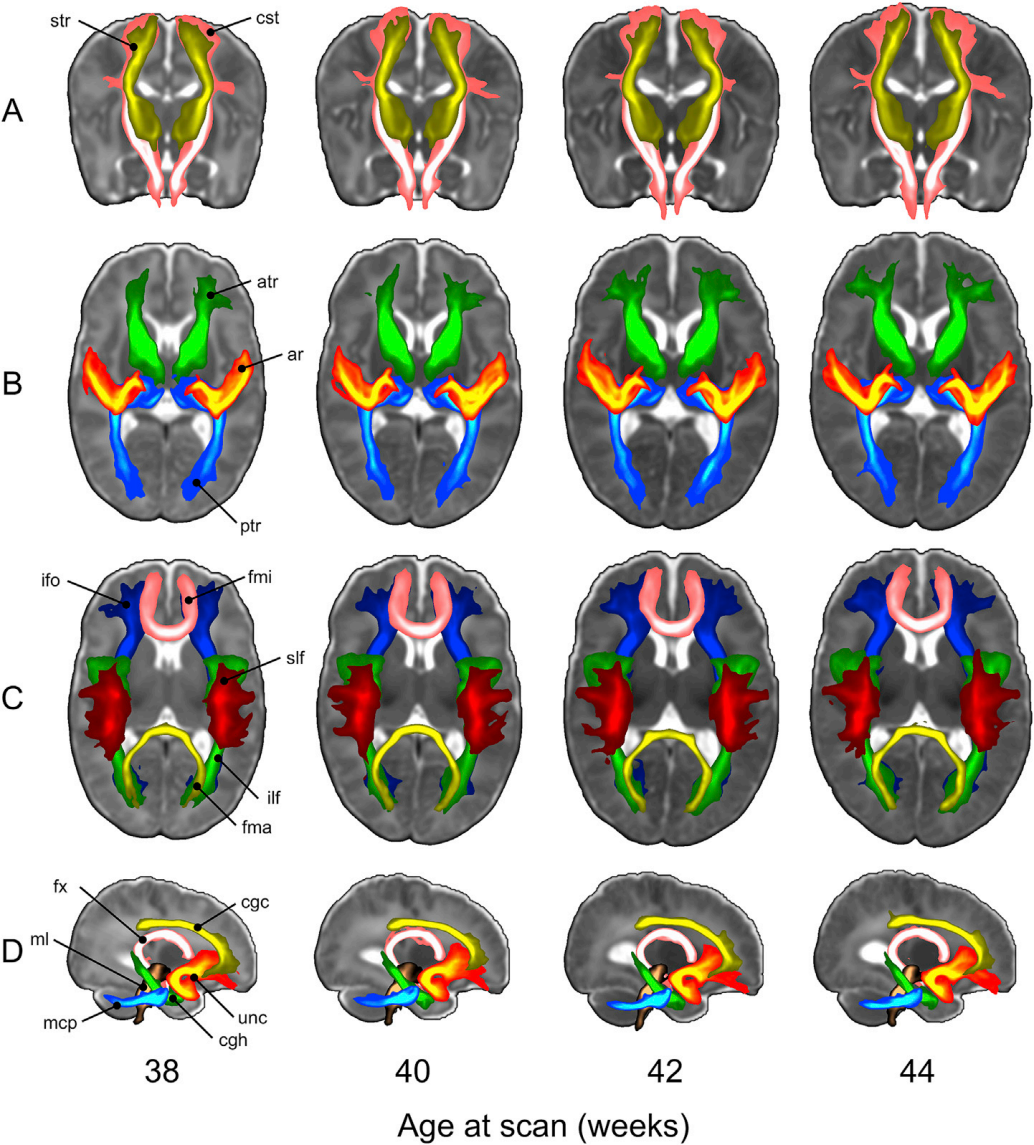
Output of the dMRI processing pipeline from 38 to 44 weeks post-menstrual age (20 subjects per bin) showing maximum intensity projections of the automated probabilistic tractography results averaged across 80 dHCP subjects (20 per age bin). All tracts are visualized using the same thresholds (0.005, 0.05). These thresholds extract the main body of the tracts and might remove lateral projections due to the variable and low number of crossings that can be estimated especially at the very young age. A) Sensory-motor projection fibres: corticospinal tract (pink) and superior thalamic radiation (yellow) B) Thalamic projection fibres: anterior thalamic radiation (green), acoustic radiation (red-yellow), posterior thalamic radiation (blue-light blue); C) Callosal and association fibres: forceps minor (pink), forceps major (yellow), superior longitudinal fasciculus (red), inferior longitudinal fasciculus (green), inferior fronto-occipital fasciculus (blue). D) Brainstem tracts and association fibres in the left hemisphere: middle cerebellar peduncle (blue-light blue), medial lemniscus (brown), cingulate gyrus part of the cingulum (yellow), parahippocampal part of the cingulum (green), fornix (pink), uncinate fasciculus (red-yellow).

Prior to starting tracking, the ROIs of each selected tract are warped to the template's time point matched to the subject's age at scan (in weeks) using non-linear registration, FNIRT (Andersson et al., 2003). This step is done to ensure that all the tracking results are stored in age-matched template space for further processing. Probabilistic tractography is then performed in subject's native diffusion space and the results are resampled from diffusion space to age-matched template space using the non-linear warp fields. When the tracking is complete, we obtain tract probability maps by normalising the resulting volumes storing the number of streamlines passing through each voxel by the total number of successful streamlines, i.e., those that were not rejected by any exclusion criterion. Finally, we average all the age-matched tract probability maps to obtain group maps. Fig. 12 shows such average tract probability maps for all the bilateral tracts. The tool can easily be extended/modified and is computationally efficient as it uses GPUs (Hernandez Fernandez et al., 2016).

**Fig. 13:**
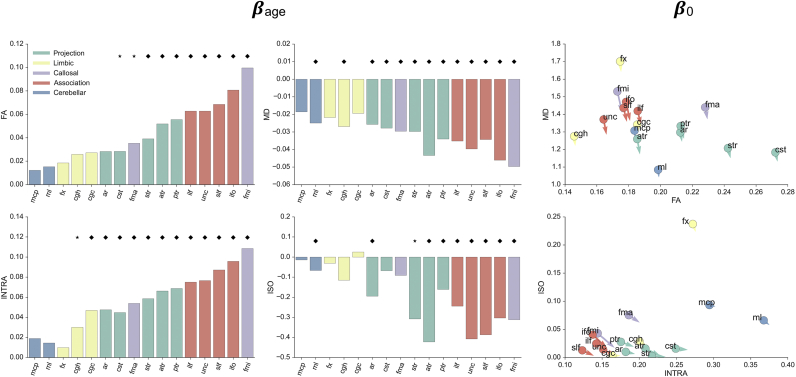
Linear regression analysis coefficients for tract-specific microstructural indices. Maturational speed (i.e., slopes) indicated as β_age_, predicted microstructural index value at the 41^st^ week (i.e., intercept) indicated as β_0_. Slopes are normalised by the tract-specific scalar value averaged across ages, to facilitate comparison. Values in bar plots are sorted according to the FA magnitudes. Diamonds indicate that microstructural measures and age-at-scan were significantly, i.e., p < 0.05 corrected related for a specific tract. Different colours represent different tracts category. Scatterplots relate microstructural indices obtained from the same analysis technique. Arrows in the scatterplot show predicted changes after one week. High MD values in the fornix can be explained (as in the case of adult MD images) by partial volume effects with CSF regions. MD is expressed in μm^2^/ms. See Table 2 for abbreviations.

Defining subject-specific tracts using tractography can be very useful for assessing tract-specific microstructural changes in the neonatal brain (Dehaene-Lambertz and Spelke, 2015; Dubois et al., 2008, 2014a; Huang et al., 2006; Lebel and Deoni, 2018; Mukherjee et al., 2002; Oishi et al., 2013; Pecheva et al., 2017; Wozniak and Lim, 2006). Most studies investigating white matter microstructure in newborns use atlas-based ROIs (Kersbergen et al., 2014) to define the core of the tracts or manually segment them (Kunz et al., 2014; Paydar et al., 2014). As an example of the use of pipeline data we used the results from the automated framework to define age-specific white matter masks to extract tract-specific microstructural indices. Fig. 13 and supplementary figure 6 show the results of a linear regression analysis relating tract-specific microstructural indices obtained from DKI and NODDI in the exemplar dataset of 140 subjects (see supplementary table 1 for a full list of regression coefficients). We added total brain volume as an extra covariate to regress out partial volume effects and de-meaned both co-variates. Tractography results were averaged across individuals in age-matched template space, thresholded at 0.01 and binarised to obtain tract-specific masks. The differences in the magnitude of the age-related regression coefficients reflect the different developmental pathways of individual white matter tracts, which is well known to be asynchronous both from post-mortem (Kinney et al., 1988; Rados et al., 2006) and in vivo dMRI studies (Dehaene-Lambertz and Spelke, 2015; Dubois et al., 2008, 2014a; Huppi and Dubois, 2006; Kersbergen et al., 2014; Kunz et al., 2014; Mukherjee et al., 2002; Oishi et al., 2011; Paydar et al., 2014).

Relating the intercepts between different microstructural indices obtained from the same analysis technique also reveals clusters based on tract category. A general increase in FA and decrease in MD with age is matched by a significant increase of intra–neurite volume fraction and a decrease of isotropic volume fraction contamination. The MD and isotropic volume fraction changes point to the generalized reduction of water content occurring in the developing brain (Neil et al., 1998).

## Discussion

In this work, we presented a comprehensive and automated pipeline to analyse neonatal dMRI data as acquired in the developing Human Connectome Project. Previous developmental studies have addressed single aspects of foetal (Oubel et al., 2012; Studholme, 2011) or neonatal data analysis, such as pre-processing (Dubois et al., 2014b; Holdsworth et al., 2012; Morris et al., 2011), microstructural modelling (Huppi et al., 1998; Kunz et al., 2014) and structural connectivity (Howell et al., 2018; Tymofiyeva et al., 2012). Our pipeline allows to analyse consistently a large number of subjects, despite the challenges that characterize neonatal dMRI data analysis (Fig. 1). We optimised existing methods and developed new ones to successfully pre-process the datasets. With a single tool, we can correct susceptibility and eddy-current-induced distortions, between (Andersson and Sotiropoulos, 2016) and within-volume (Andersson et al., 2017) motion artefacts and signal dropouts (Andersson et al., 2016). Moreover, we introduced an automated QC framework that allows to detect issues and data inconsistencies occurring at different stages of the pipeline and to quantify overall data quality. Such a framework is crucial when dealing with big cohorts of largely non-compliant subjects that make manual QC very time consuming and subjective.

The neonatal brain is not just a miniature adult brain (Batalle et al., 2017a), but poses several analysis challenges. The dMRI signal profile is very different when compared to the adult. Newborns are characterised by higher water content and lower myelination. Moreover, grey and white matter also show different microstructural features. Previous work argued that using a low b-value (∼700 s/mm^2^) might be optimal for neonatal dMRI due to the higher water content (Pannek et al., 2012). For the dHCP, we have used a data-driven approach to determine the optimal combination of diffusion encoding orientations and b-values (Tournier et al., 2015; Tournier et al., 2018, under revision). This allowed us to maximise the contrast between different tissue types and to use advanced analysis techniques that can estimate complex fibre geometries.

Furthermore, newborns typically show higher levels of motion when compared to adult subjects. Artefacts that are not so noticeable in adults become prominent in neonates, such as within-volume (sudden) motion, slice misalignment, and signal dropouts. The gyrification pattern, contrast and head sizes vary significantly throughout development. Therefore, we have developed new approaches that address such bespoke scenarios. Additionally, we used age-specific templates and transformations, as well as a specialized MR coil that improves SNR when imaging small brains.

Most previous studies have used DTI to assess microstructural changes and estimate structural connectivity profiles. The data quality and acquisition strategy followed in the dHCP allow the use of more advanced multi-shell analysis techniques, which in principle can estimate richer microstructural features and multiple fibre orientations within the same voxel. To realise these benefits in tractography, we used an approach for estimating fibre crossings, which accounts for the low anisotropy of the neonatal brain by assuming a non-perfectly anisotropic response function. This is crucial when estimating fibre orientations using a spherical deconvolution approach, which typically assume the same deconvolution kernel across the whole brain (Parker et al., 2013). This assumption does not hold in the neonatal brain, where localised changes in anisotropy occur throughout development due to changes in myelination and development of cortico-cortical projections. We therefore utilised an approach that relaxes this assumption and allows variations of the deconvolution kernel through space.

Using the voxel-wise estimated fibre orientations, we developed a neonatal-specific automated tractography tool. This allows to automatically segment white matter into a set of subject-specific long-range projections. These can be used to define regions of interests for further tract-specific microstructural or morphological analyses.

All the data used in this work will be shared with the scientific community. Raw and processed dMRI, structural and rfMRI datasets will be made available at http://www.developingconnectome.org as part of the official dHCP public data releases. This includes atlases and ROIs for, e.g., automated tractography. Processing pipelines will also be made available on the same platform. The current version of the dHCP neonatal dMRI pipeline can already be downloaded from: https://git.fmrib.ox.ac.uk/matteob/dHCP_neo_dMRI_pipeline_release.

In this work, we have described the current version of the dMRI neonatal processing pipeline. However, there are ongoing developments to address additional challenges in the data analysis stream. For instance, because of the high degree of motion present in neonatal data, the susceptibility field can change significantly during the acquisition. This means that a single fieldmap estimate is not sufficient and should be updated in the presence of big rotations and translations (Andersson et al., 2018). Moreover, registration to anatomical space can be improved. Diffusion MRI data provides additional information when compared to structural acquisitions (e.g., T1 or T2-weighted). This can be used to improve the registration to subject's anatomical space using, e.g., the diffusion tensor (Zhang et al., 2006). Tractography results can also be improved by using methods that allow the estimation of complex asymmetric sub-voxel fibre patterns (Bastiani et al., 2017). Both cortical microstructure and connectome reconstruction can benefit from improved surface registration, such as advanced techniques that allow to register surfaces based on multiple modalities (Robinson et al., 2013), together with templates that preserve cortical locations across ages.

## Conclusions

We have presented an automated pipeline to pre-process neonatal dMRI data that is capable of effectively dealing with the main challenges presented by neonatal data and providing high-quality distortion correction, brain microstructure and connectivity results across early developmental ages, and provided exemplars of the output of the pipeline and the use of the data. The pipeline will allow researchers to use the dHCP datasets to explore important questions about the development of the human brain.

## Appendix A Supplementary data

SupplementaryMaterial.pdf SupplementaryMaterial.pdf
