# Supplementary material for: Automated processing pipeline for neonatal diffusion MRI in the developing Human Connectome Project
Source: Neuroimage. 2019 Jan 15;185:750–63. doi: 10.1016/j.neuroimage.2018.05.064 (PMC6299258; doi:10.1016/j.neuroimage.2018.05.064)
Supplement: SupplementaryMaterial.pdf [file mmc1.pdf]

Automated processing pipeline for neonatal diffusion MRI in the developing Human Connectome Project

Authors:

Matteo Bastiani[1], Jesper Andersson[1], Lucilio Cordero-Grande[2], Maria Murgasova[2], Jana Hutter[2], Anthony N. Price[2], Antonios Makropoulos[3], Sean P. Fitzgibbon[1], Emer Hughes[2], Daniel Rueckert[3], Suresh Victor[2], Mary Rutherford[2], A. David Edwards[2], Steve Smith[1], Jacques-Donald Tournier[2], Joseph V. Hajnal[2], Saad Jbabdi[1], Stamatios N. Sotiropoulos[1,4]

[1] Wellcome Centre for Integrative Neuroimaging - Oxford Centre for Functional Magnetic Resonance Imaging of the Brain (FMRIB), University of Oxford, UK
[2] Centre for the Developing Brain, King's College London, UK
[3] Department of Computing, Imperial College London, UK
[4] Sir Peter Mansfield Imaging Centre, School of Medicine, University of Nottingham, UK

Supplementary Material

Supplementary figures

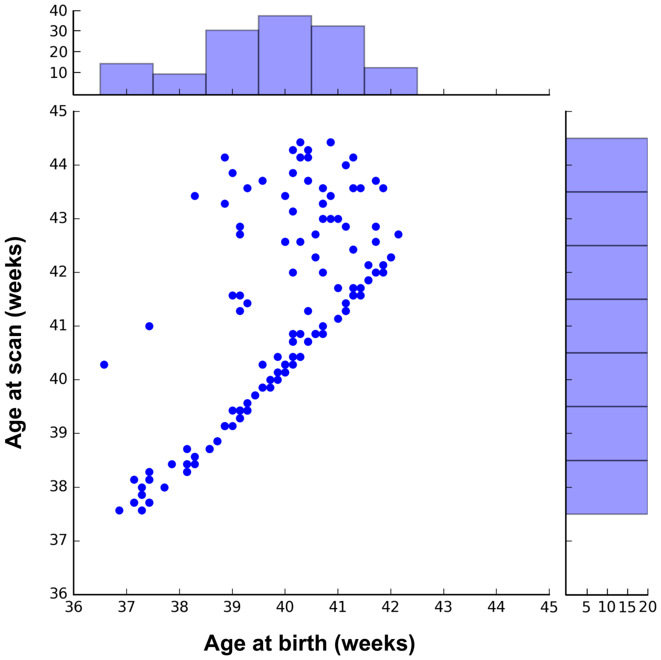

Supplementary figure 1: Age at birth and at scan distributions for the 140 subjects used in this work. The scatter plot shows the individual relation between age at scan and age at birth. Most neonates were scanned shortly after birth, with a median difference between age at scan and age at birth of 0.5 weeks.

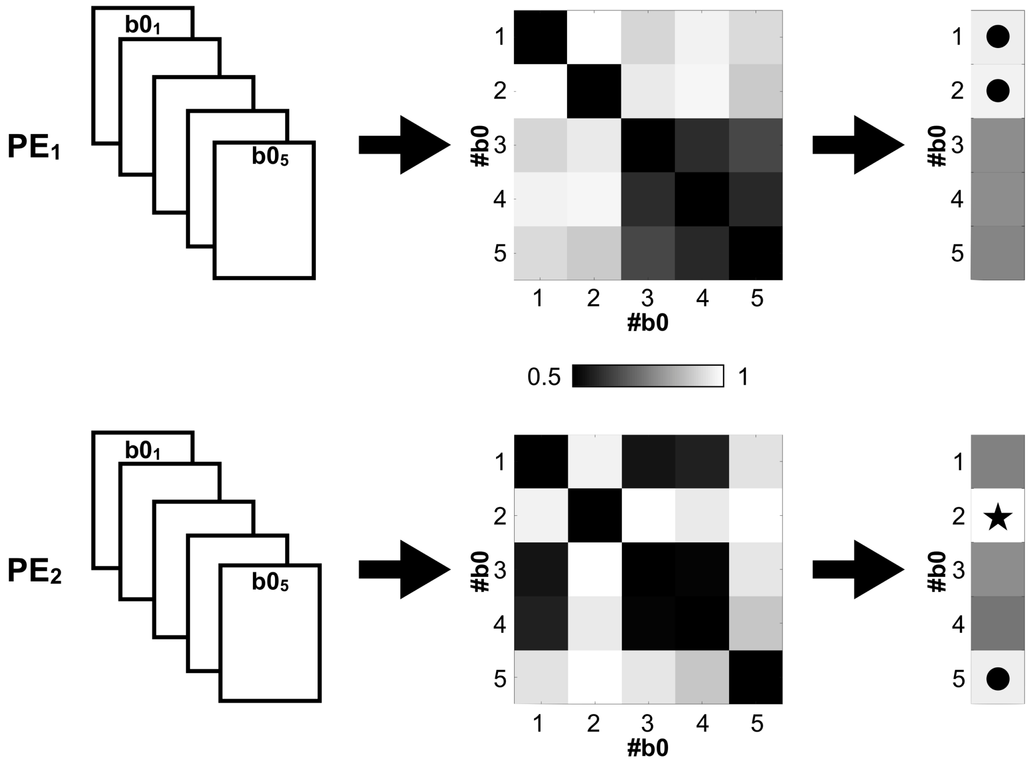

Supplementary figure 2: Selection of the best b0 volumes and reference scan. For each PE direction, the correlations between each pair of b0 volumes are computed after rigid alignment (6 DOFs). The b0 volumes showing the highest average correlation (i.e., excluding the diagonal values) are then chosen (black dots). The b0 volume with the highest correlation amongst all acquired PE directions is chosen as the reference volume (black star).

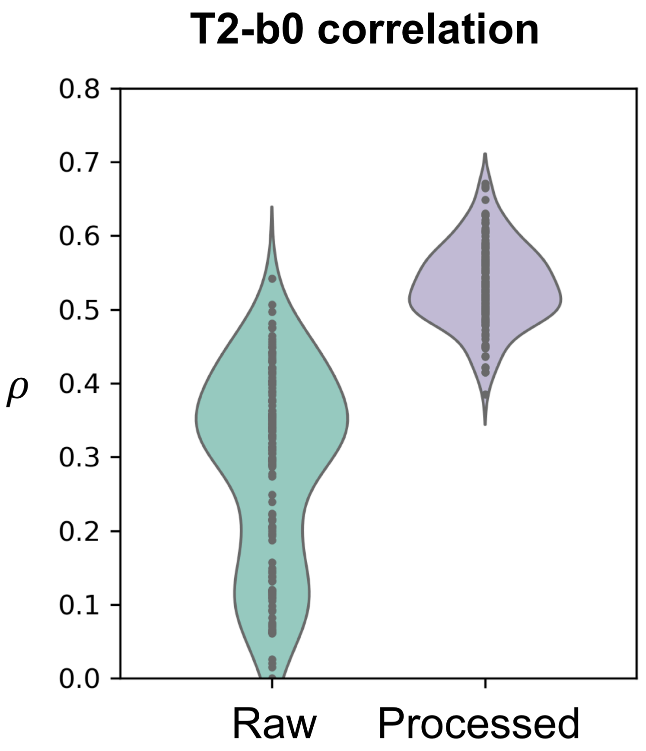

Supplementary figure 3: Quantitative assessment of processing and registration performances. The correlation between the high-resolution T2-weighted volume and the averaged b0 volume shows a significant improvement between processed and raw data (median correlations for 140 at term newborns: 0.33 for raw and 0.53 for processed data).

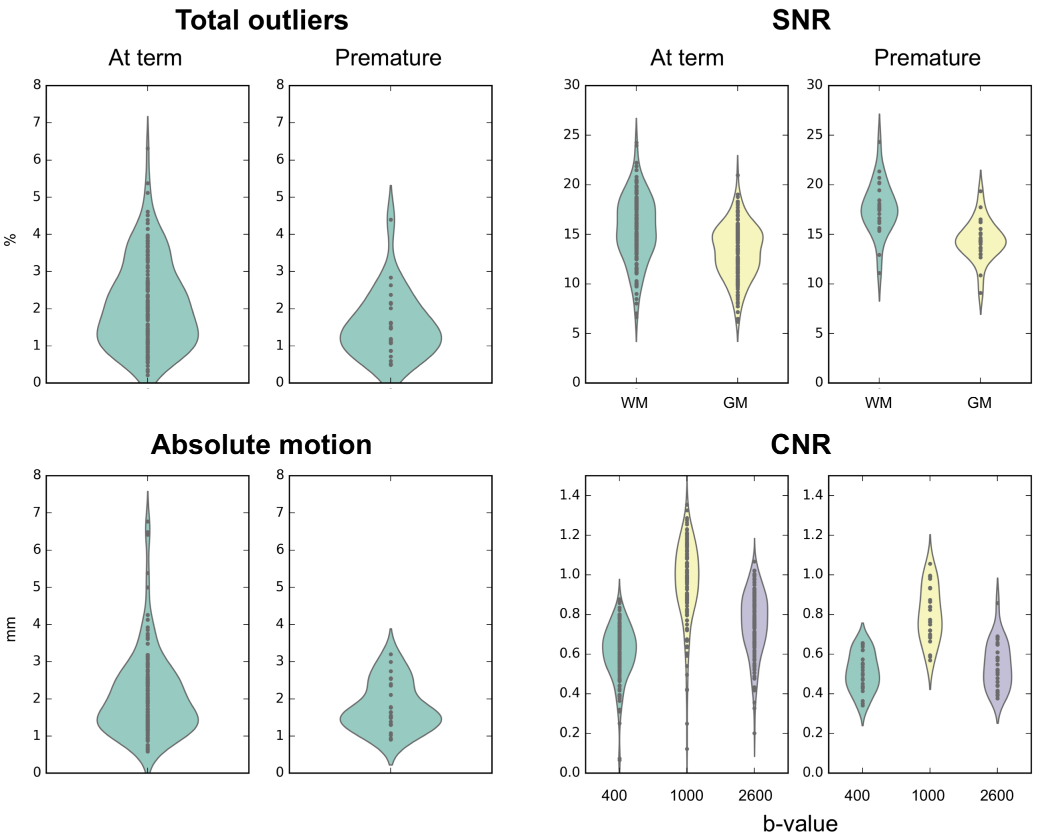

Supplementary figure 4: QC metrics comparison between preterm and born at term infants. The distributions were derived from the 140 at term subjects used throughout the article and from 25 infants born preterm (age at scan range: 29 to 35 weeks. Median values and distributions overlap nicely for total outliers, average motion and SNR in both tissue types. Diffusion CNR values are slightly lower in the preterm infants because of a reduced anisotropy and higher water content in the brain.

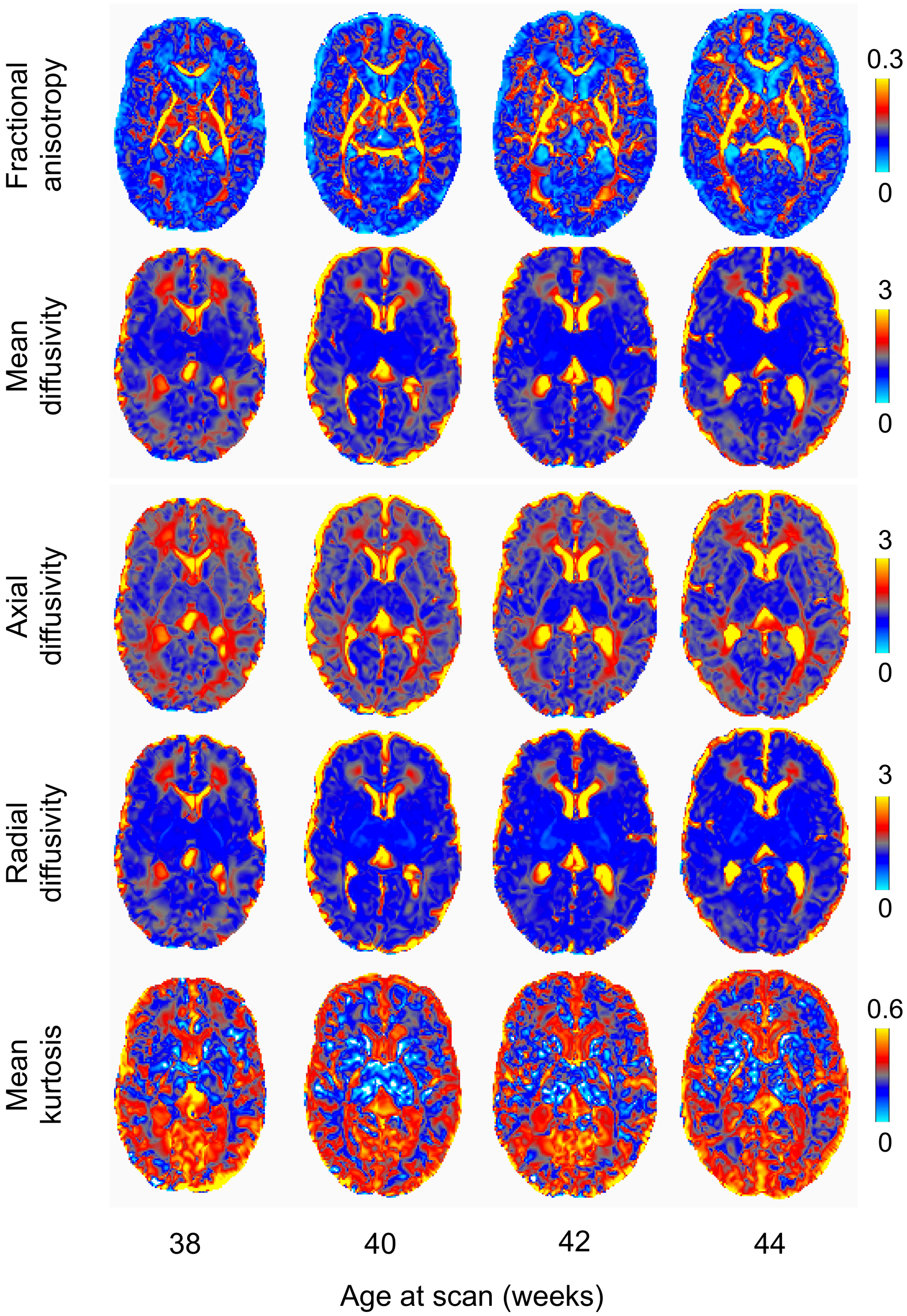

Supplementary figure 5: Output of the dMRI processing pipeline showing single-subject DT-derived microstructural measures from 38-44 weeks. Diffusivities are expressed in $\mu m^2/ms$.

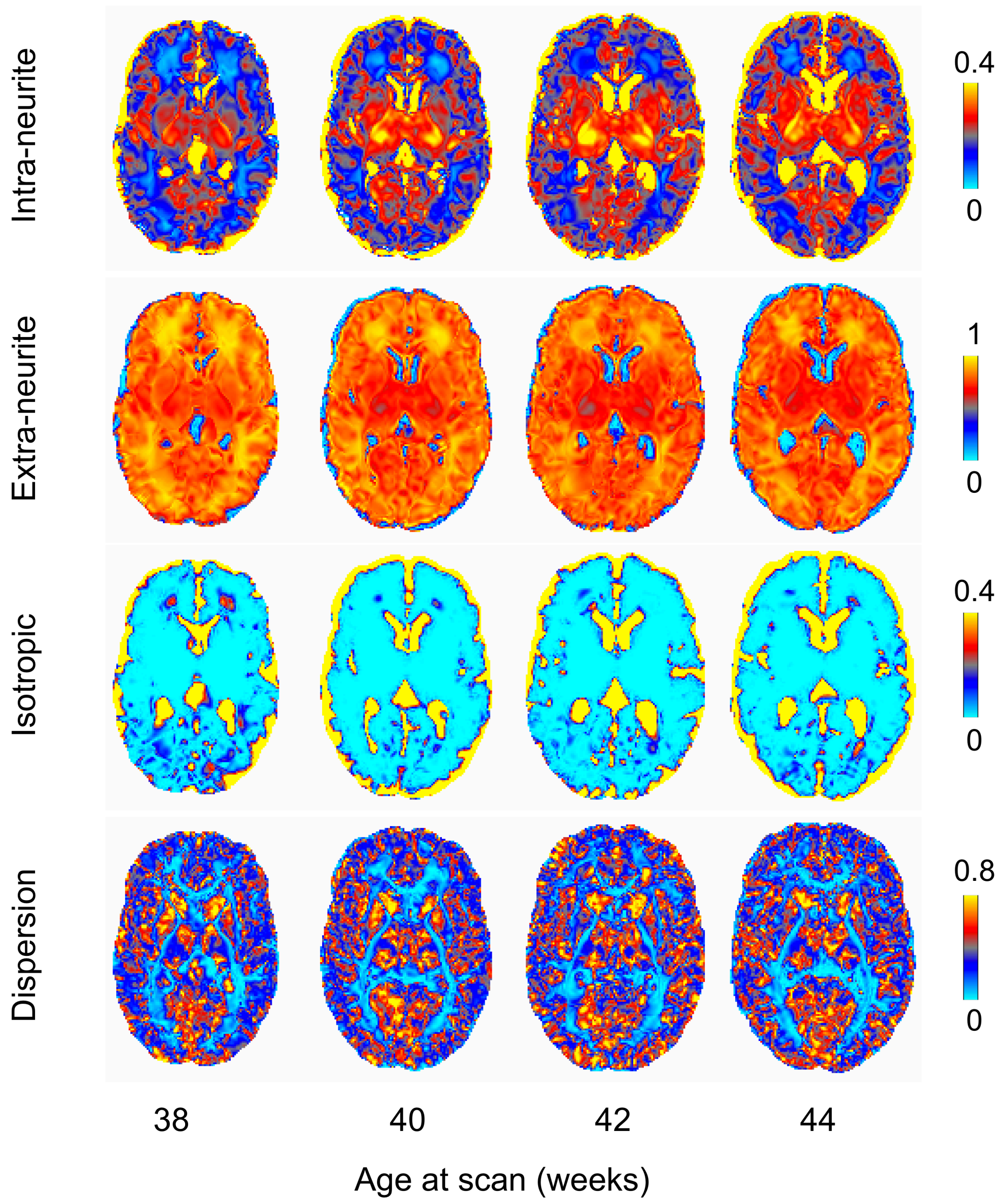

Supplementary figure 6: Output of the dMRI processing pipeline showing single-subject NODDI metrics from 38 to 44 weeks post-menstrual age.

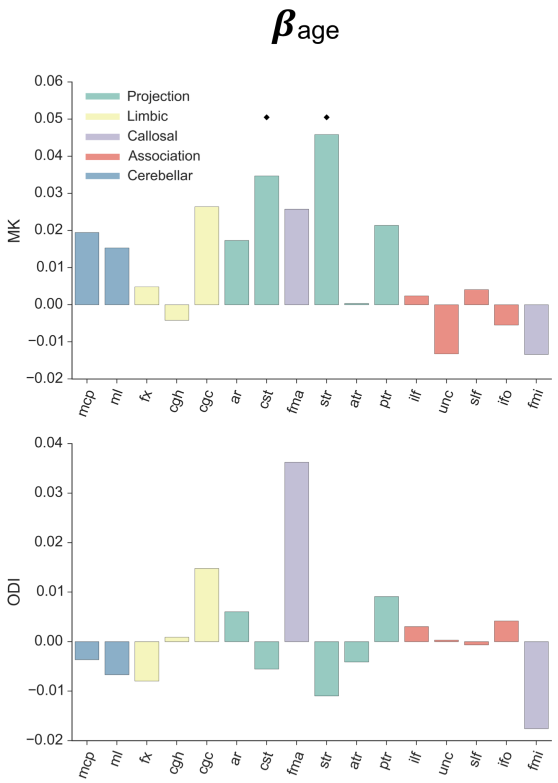

Supplementary figure 7: Linear regression analysis coefficients for tract-specific microstructural indices (MK and ODI). Maturational speed (i.e., slopes) indicated as β_{age}. Slopes are normalised by the tract-specific scalar value averaged across ages, to facilitate comparison. Values in bar plots are sorted according to the FA magnitudes. Diamonds indicate that microstructural measures and age-at-scan were significantly, i.e., $p < 0.05$ corrected related for a specific tract. See table 2 in the main text for abbreviations.

Supplementary tables

Index	Tracts	β_0	β_{age}	β_{tbv}
FA	ar	0.2124	0.0060	-0.0001
	atr	0.1854	0.0096**	-0.0002
	cgc	0.1854	0.0050	-0.0002
	cgh	0.1458	0.0038	-0.0001
	cst	0.2722	0.0077*	-0.0002
	fma	0.2281	0.0081*	-0.0003
	fmi	0.1729	0.0172**	-0.0003
	fx	0.1746	0.0032	-0.0002
	ifo	0.1783	0.0144**	-0.0002
	ilf	0.1857	0.0117**	-0.0002
	mcp	0.1837	0.0022	-0.0001
	ml	0.1985	0.0030	0.0001
	ptr	0.2127	0.0118**	-0.0002
	slf	0.1768	0.0121**	-0.0003
	str	0.2423	0.0095**	-0.0003
	unc	0.1642	0.0103**	-0.0002

MD	ar	0.0013	0.0000**	0.0000
	atr	0.0013	-0.0001**	0.0000**
	cgc	0.0013	0.0000	0.0000
	cgh	0.0013	0.0000**	0.0000
	cst	0.0012	0.0000**	0.0000
	fma	0.0014	0.0000**	0.0000
	fmi	0.0015	-0.0001**	0.0000*
	fx	0.0017	0.0000	0.0000**
	ifo	0.0015	-0.0001**	0.0000
	ilf	0.0014	0.0000**	0.0000
	mcp	0.0013	0.0000	0.0000
	ml	0.0011	0.0000**	0.0000
	ptr	0.0013	0.0000**	0.0000
	slf	0.0014	0.0000**	0.0000
	str	0.0012	0.0000**	0.0000*
	unc	0.0014	-0.0001**	0.0000
MK	ar	0.2759	0.0048	-0.0001
	atr	0.2832	0.0001	0.0002
	cgc	0.2423	0.0064	0.0002
	cgh	0.2794	-0.0012	0.0001
	cst	0.3433	0.0119**	-0.0001
	fma	0.3238	0.0083	-0.0001
	fmi	0.3142	-0.0042	0.0002
	fx	0.3950	0.0019	0.0001
	ifo	0.2870	-0.0016	0.0001
	ilf	0.2766	0.0007	0.0001
	mcp	0.4316	0.0084	0.0000
	ml	0.4543	0.0070	-0.0003
	ptr	0.2796	0.0060	0.0000
	slf	0.2221	0.0009	0.0001
	str	0.2979	0.0137**	-0.0001
	unc	0.2546	-0.0034	0.0000
INTRA	ar	0.1817	0.0086**	-0.0002
	atr	0.2082	0.0138**	-0.0003
	cgc	0.1636	0.0077**	-0.0002
	cgh	0.2004	0.0060*	0.0000
	cst	0.2491	0.0111**	-0.0002
	fma	0.1860	0.0100**	-0.0002
	fmi	0.1440	0.0156**	-0.0002
	fx	0.2720	0.0027	0.0006*
	ifo	0.1373	0.0132**	-0.0002
	ilf	0.1421	0.0107**	-0.0002
	mcp	0.2944	0.0056	0.0001
	ml	0.3675	0.0053	-0.0001
	ptr	0.1752	0.0120**	-0.0002

	slf	0.1233	0.0108**	-0.0003*
	str	0.2171	0.0127**	-0.0004**
	unc	0.1513	0.0116**	-0.0002
ISO	ar	0.0101	-0.0020	0.0000
	atr	0.0160	-0.0067	0.0002**
	cgc	0.0033	0.0001	0.0000
	cgh	0.0283	-0.0033	0.0002
	cst	0.0158	-0.0011	0.0001
	fma	0.0752	-0.0068	0.0003
	fmi	0.0431	-0.0135**	0.0004*
	fx	0.2373	-0.0073	0.0010**
	ifo	0.0407	-0.0123**	0.0004**
	ilf	0.0248	-0.0061**	0.0002*
	mcp	0.0932	-0.0013	0.0001
	ml	0.0661	-0.0044**	0.0000
	ptr	0.0278	-0.0045**	0.0002**
	slf	0.0128	-0.0049**	0.0001*
	str	0.0027	-0.0008*	0.0000
	unc	0.0143	-0.0058**	0.0001
ODI	ar	0.1706	0.0010	0.0000
	atr	0.2508	-0.0010	-0.0002
	cgc	0.1999	0.0030	-0.0001
	cgh	0.3193	0.0003	0.0001
	cst	0.1509	-0.0008	0.0000
	fma	0.1211	0.0044	0.0000
	fmi	0.1212	-0.0021	0.0000
	fx	0.1466	-0.0012	0.0001
	ifo	0.1220	0.0005	0.0000
	ilf	0.1260	0.0004	0.0000
	mcp	0.2384	-0.0009	0.0002
	ml	0.2920	-0.0019	-0.0001
	ptr	0.1476	0.0013	0.0000
	slf	0.1550	-0.0001	0.0001
	str	0.1728	-0.0019	0.0000
	unc	0.1733	0.0001	0.0000

Supplementary table 1: Linear regression coefficients for tract-specific microstructural analysis. **: $p < 0.01$ (Bonferronicorrected), *: $p < 0.05$ (Bonferroni corrected).
